# Extracellular Optogenetics at the Interface of Synthetic Biology and Materials Science

**DOI:** 10.3389/fbioe.2022.903982

**Published:** 2022-06-14

**Authors:** Lisa K. Månsson, Angela A. Pitenis, Maxwell Z. Wilson

**Affiliations:** ^1^ Materials Department, University of California, Santa Barbara, Santa Barbara, CA, United States; ^2^ Center for BioEngineering, University of California, Santa Barbara, Santa Barbara, CA, United States; ^3^ Department of Molecular, Cellular, and Developmental Biology, University of California, Santa Barbara, Santa Barbara, CA, United States; ^4^ Neuroscience Research Institute, University of California, Santa Barbara, Santa Barbara, CA, United States

**Keywords:** light-sensitive proteins, programmable materials, biocompatible materials, dynamic extracellular matrix, organoids

## Abstract

We review fundamental mechanisms and applications of OptoGels: hydrogels with light-programmable properties endowed by photoswitchable proteins (“optoproteins”) found in nature. Light, as the primary source of energy on earth, has driven evolution to develop highly-tuned functionalities, such as phototropism and circadian entrainment. These functions are mediated through a growing family of optoproteins that respond to the entire visible spectrum ranging from ultraviolet to infrared by changing their structure to transmit signals inside of cells. In a recent series of articles, engineers and biochemists have incorporated optoproteins into a variety of extracellular systems, endowing them with photocontrollability. While other routes exist for dynamically controlling material properties, light-sensitive proteins have several distinct advantages, including precise spatiotemporal control, reversibility, substrate selectivity, as well as biodegradability and biocompatibility. Available conjugation chemistries endow OptoGels with a combinatorially large design space determined by the set of optoproteins and polymer networks. These combinations result in a variety of tunable material properties. Despite their potential, relatively little of the OptoGel design space has been explored. Here, we aim to summarize innovations in this emerging field and highlight potential future applications of these next generation materials. OptoGels show great promise in applications ranging from mechanobiology, to 3D cell and organoid engineering, and programmable cell eluting materials.

## 1 Introduction

Photoswitchable proteins, or optoproteins, unlock the precise spatiotemporal control of programmable light delivery devices developed in other industries (e.g., projectors, lasers, LEDs, and lithography) through their ability to sense light and respond by changing their conformational structure (Tischer and Weiner (2014); Mansouri et al. (2019); Lu et al. (2020)). While their applications in biology and neuroscience have recently exploded, research into their structure has captivated scientists for over 50 years.

**FIGURE 1:**
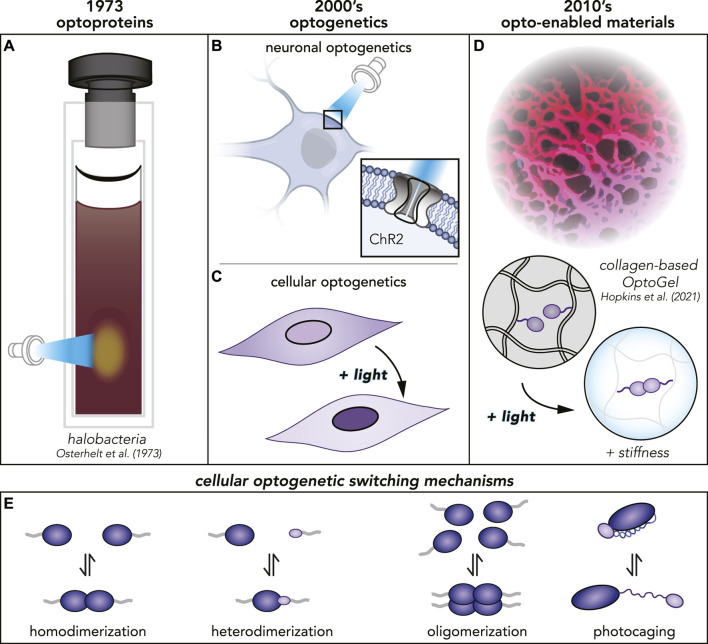
**(A)** The first optoprotein found in halobacteria reported 1973. **(B, C)** Several decades later, many more optoproteins had been discovered and the field of optogenetics was born. Optoproteins are used in neuronal as well as cellular optogenetics with different applications. **(D)** Today, optoproteins have been purified and proven to function even in materials. **(E)** Different photoswitching mechanisms for different optoproteins used in cellular optogenetics.

The first optoproteins were discovered serendipitously in 1973, when researchers noticed reversible, light-responsive chromographic changes in membrane isolates from halobacteria (Oesterhelt and Stoeckenius (1973)) (Figure 1A). Decades later, neuroscience was revolutionized with the discovery that engineering algal Channelrhodopsins (*ChR2*) into mammalian neurons endowed them with light-responsive control of action potentials (Boyden et al. (2005); Deisseroth (2015)) (Figure 1B). This clever recontextualization of a photosensitive molecule, fine-tuned by evolution in another context (the algal cytoplasm), is the canonical example of how optoproteins can be a key enabling technology for endowing complex systems with photocontrollability. Since the early 2000s (Shimizu-Sato et al. (2002); Boyden et al. (2005)), the optoprotein toolbox has been expanded, beyond the neuron, into controlling a wide variety of biological processes (Tischer and Weiner (2014); Mansouri et al. (2019); Burgos-Morales et al. (2021)), such as cell differentiation (Krishnamurthy et al. (2016)), migration (Wu et al. (2009a)), signaling (Reichhart et al. (2016)), apoptosis (Mills et al. (2011)), and even gene editing (Nihongaki et al. (2015)). The use of optoproteins to control biological processes in the cell cytoplasm is termed cellular optogenetics (Figure 1C).

Optoproteins are poised to revolutionize materials science by enabling the programmatic interrogation of material properties. Experiments requiring biochemical reconstitution to test for the sufficiency of the molecular constituents in phototropism and photoentrainment proved that optoproteins could photoswitch robustly in test tubes (Butler et al. (1959); Malhotra et al. (1994); Ahmad et al. (1998); Christie et al. (1999)). This discovery underlies a recent flurry of investigations probing the application space of optoproteins completely outside of the cell.

Conventional biomaterials have mainstream uses ranging from wound dressing, to cell culture substrates, to medical device coatings, but have historically lacked dynamic spatiotemporal control (Ahmad et al. (2022)). Besides their uses in materials, optoproteins have been used in a variety of other extracellular contexts that are outside of the scope of this review. Some examples include their use in light-controlled viral transduction (Hörner et al. (2021)), light-controlled cell-cell assembly (Mombo et al. (2021)), and bacteria-driven microswimmers (Sentürk et al. (2020)). Here, we focus on a branch of extracellular optogenetics which encompasses biochemical syntheses of new, dynamic materials and integration of biological components into synthetic or natural polymer networks (Figure 1D). The interface between Synthetic Biology and Materials Science poses a promising new Frontier with a wide range of potential applications. As we approach the 50th anniversary since the discovery of optoproteins, we highlight cutting-edge research and look forward to new uses of these molecules within the class of Opto-Enabled Materials.

## 2 Opto-Enabled Materials: A Vast Design Space

Many routes exist for dynamically controlling material properties (e.g., pH-, temperature-, light-responsiveness). Optoproteins have several distinct advantages over synthetic photoswitch chemistries, including biodegradability and bioorthogonality. To date, thousands of optoproteins have been identified in the genomes of microorganisms (Glantz et al. (2016)), each likely with unique properties. Over billions of years of evolution, optoproteins have acquired a wide spectrum of light-responsive mechanisms (Figure 1E) (Mansouri et al. (2019); Lu et al. (2020); Wu et al. (2009b); Baumschlager and Khammash (2021); Levskaya et al. (2009); Kennedy et al. (2010); Zhang et al. (2017)). In contrast to many synthetic chemistry routes, optoproteins are not petroleum-based, compatible with aqueous environments, and require low ambient intensities of light to photoswitch (Hörner et al. (2019); Hopkins et al. (2021); Liu L. et al. (2018); Duan et al. (2021); Narayan et al. (2021); Wang et al. (2017)).

**FIGURE 2:**
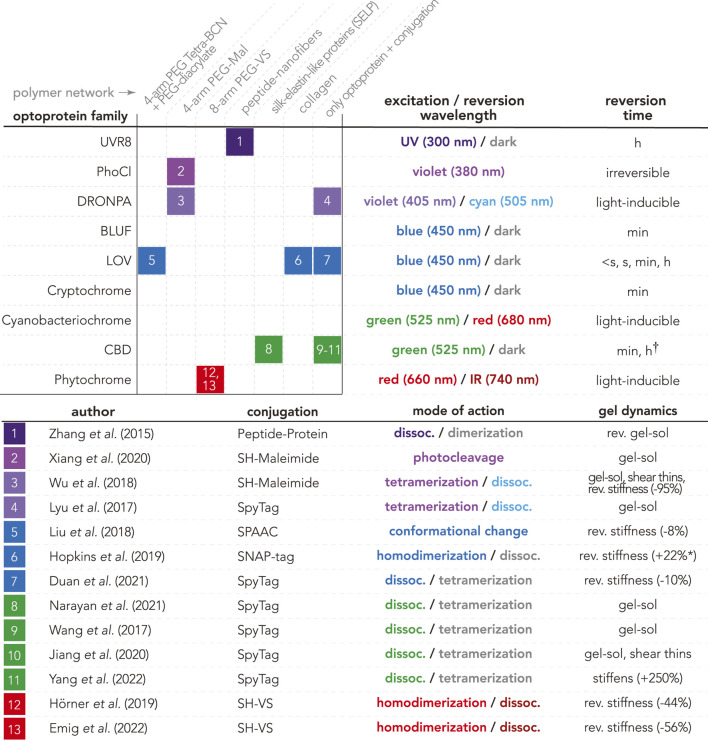
OptoGel design space, including OptoGels presented in the literature based on combinations of optoproteins (rows) and polymer networks (columns). Each box is representing the OptoGel(s) referenced in the enumerated list, including information about conjugation chemistry used, mode of action (colored as the excitation/reversion wavelength, or gray for dark), as well as reported dynamic light-tunable material properties for the gel. Dynamic ranges of stiffness from AFM measurements of elastic modulus E are marked with *, and those from rheometry measurements of storage modulus G′ are unmarked. Note that non-reported properties can mean that it was just not tested. Optoprotein data from Optobase (excitation/reversion wavelengths and reversion times) (Kolar et al. (2018)), and from (Hu et al. (2020)) marked with †.

Purified optoproteins have recently been incorporated into opto-enabled materials to form “OptoGels” (Figure 2). An OptoGel is a hydrogel with light-programmable properties dynamically crosslinked by optoproteins. The first OptoGel was made in 2015 by Zhang *et al.* with UV-B resistance 8 (UVR8) optoproteins linking networks of nanofibers and peptides (Zhang et al. (2015)). Despite the vast landscape of design parameters, relatively few OptoGels have been made since, as can be seen in Figure 2. This section will discuss OptoGel design space and trends, as well as their physical properties and dynamic ranges.

Another class of opto-enabled materials use opsin-based optoproteins, which are light-controlled membrane-based ion-channels (Figure 1B). These opsin-based opto-enabled materials have been used over the past 40 years in photosensing applications, including optical information carriers and holographic interferometric devices (Hampp (2000)). Continuing research efforts have focused on their utility in sensors, catalysts, and systems for controlled ion transport (Jahnke et al. (2018); Gaitzsch et al. (2019)). Opsin-based optoproteins are beyond the scope of the non-opsin-based opto-enabled materials discussed herein.

### 2.1 OptoGel Design Space and Trends

OptoGels are generally composed of optoproteins and polymer networks. Figure 2 presents a map of the currently known optoprotein/polymer network combinations along with their conjugation chemistries as well as observed light-controlled material properties (e.g. tunable stiffness, reversibility, and phase transitions). There are three optoprotein families that have been well-characterized *in vitro* yet are entirely absent in the current OptoGel design space (empty rows in Figure 2): blue-light-using flavin (BLUF) domains, cryptochromes, and cyanobacteriochromes. These families may be absent due to their biochemical peculiarities. For example: cryptochromes form dynamic clusters *in vivo* (Che et al. (2015)) but are difficult to purify *in vitro* and often crash out of solution. We anticipate that overcoming the experimental and handling challenges of purifying cryptochromes would open up new opportunities for controlling phase transitions in opto-enabled materials, further discussed in Section 2.2. Thus, there is a vast unexplored design space with opportunities for new OptoGels solely considering which optoproteins could be incorporated.

To date, most OptoGels feature either synthetic or natural polymer networks; the remainder are gels consisting of crosslinked optoproteins without additional polymer networks. A variety of polyethylene glycol (PEG) molecular weights (ranging from 3.5 to 40 kDa) and architectures (e.g., linear, 4-arm, 8-arm) have been used to create synthetic OptoGel networks. Natural polymer networks have also been used to create OptoGels, including: self-assembling peptides, silk-elastin-like proteins (SELPs), and collagen. However, there remains a rich diversity of both synthetic and natural polymers for future investigations. The current design space of OptoGels presented in Figure 2 does not only aim to show what has been done, but to also reveal the large unexplored space of possible materials with yet unknown features.

Limited experimental data have restricted predictive modeling capabilities for opto-enabled material properties. The recent emergence of machine learning and artificial intelligence tools within biology (e.g., bioinformatics algorithms (Glantz et al. (2016)), AlphaFold (Jumper et al. (2021))) could enable the rapid discovery of new application-specific optoproteins. However, the complex interactions between dynamic optoproteins and their surroundings in the bulk (e.g., network strands, solvent molecules, other optoproteins) necessitate experiments across multiple length- and time-scales. To date, the literature contains a variety of optoproteins, networks, and experimental conditions, which both frustrates fundamental comparisons across different material systems, yet illuminates a wide open design space. Efforts to aggregate data on optoproteins and their applications within cellular optogenetics have culminated in the Optobase platform (Kolar et al. (2018)). However, design tools to predict macroscale properties from constituents of opto-enabled materials are currently lacking. Thus, the 1) standardization of opto-enabled materials characterization and 2) exploration of their combinatorially large design space present fruitful opportunities to advance the field.

### 2.2 Physical Properties and Characterization Techniques

Many physical properties (e.g., structure, mode of action) of model optoproteins are well-documented (Kolar et al. (2018)), yet optoproteins impart materials with unique physical properties that can be investigated using a multitude of techniques. There are a number of techniques and measurements that are well suited to characterize the wide varieties of observed light-controlled material properties (see Figure 2, column 4).

**FIGURE 3:**
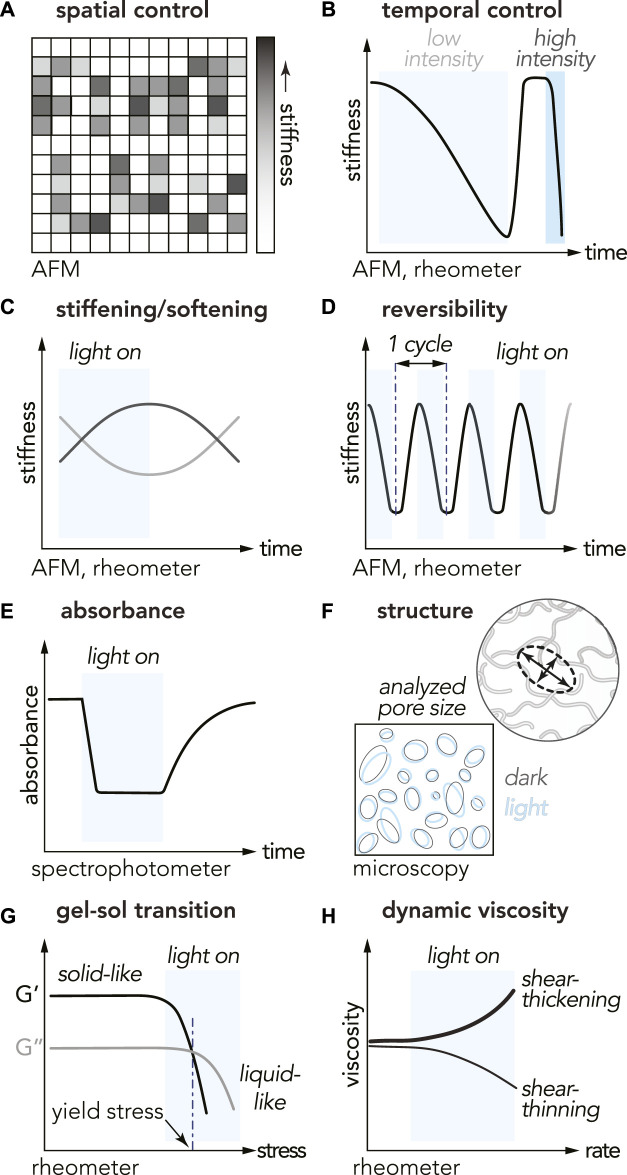
Physical properties and characterization methods for studying opto-enabled materials. The cartoons illustrate characteristic trends observed with different OptoGels **(A)** spatial control of stiffness **(B)** temporal control with light intensity dependence for the time of switch **(C)** stiffness tuning with light ON/OFF: stiffening (black curve) or softening (gray curve) in light **(D)** reversibility over multiple ON/OFF cycles **(E)** decreased absorbance during light-activation of optoprotein **(F)** material pore size comparison between light and dark states **(G)** gel-sol transition, and **(H)** shear thinning/thickening measurements.

Spatial (Wu et al. (2018); Hörner et al. (2019); Liu L. et al. (2018)) and temporal (Hörner et al. (2019); Wu et al. (2018); Liu L. et al. (2018); Narayan et al. (2021); Jiang et al. (2020); Xiang et al. (2020); Wang et al. (2017); Hopkins et al. (2021); Duan et al. (2021); Yang et al. (2022); Emig et al. (2022)) control of OptoGel stiffness has been demonstrated using atomic force microscopy (AFM) and/or rheology (Figure 3A,B). Two commonly reported rheological quantities are storage modulus (*G*′) and loss modulus (*G* ^″^) describing elastic and viscous responses of a material, respectively. The storage modulus acts as a measurement of material stiffness. In AFM measurements, the stiffness of a material is quantified by the elastic modulus (*E*). The elastic modulus is related to the storage modulus via *E* = 2*G*′(1 + *ν*), where *ν* is the Poisson’s ratio which describes how a material deforms under a load (Kollár and Tarján (2021)). Comparison of physical properties, such as stiffness, is not always trivial between different measurements on different materials. OptoGels have shown light-induced spatiotemporal control of stiffness, and that their response varies between different material designs.

A measure of stiffness tunability is the dynamic range, here defined as the ratio of the change in stiffness and the initial stiffness, yielding $\Delta G^{\prime }/G_{0}^{\prime }$ for storage modulus measurements, and Δ*E*/*E*_0_ for elastic modulus. In one example, OptoGels composed of polyethylene glycol (PEG) functionalized with phytochrome-like protein Cph1 (Cph1) optoproteins exhibited repeatable, reversible, and tunable changes in stiffness (dynamic range = 44%) in response to light (Figures 3C,D) (Hörner et al. (2019)). Photorheometry has also been used to characterize the temporal cycling of OptoGel stiffness (Liu L. et al. (2018)). Combined, these characterization techniques have revealed that baseline OptoGel stiffness and dynamic ranges in stiffness changes between the light and dark states are governed by the concentration of optoproteins embedded in the gel network (Hörner et al. (2019); Xiang et al. (2020); Duan et al. (2021); Liu L. et al. (2018); Hopkins et al. (2021); Wu et al. (2018); Lyu et al. (2017)). Estimated dynamic ranges on the OptoGels are wide spread (see Figure 2) and we currently don’t understand what fundamental properties of OptoGels contribute to this.

Reversible mechanical and rheological properties offer additional dimensions of light-programmable control. Reversibility with negligible hysteresis has been reported for many OptoGels, though the number of ON/OFF cycles tested has ranged from less than five to over 100 (Zhang et al. (2015); Wu et al. (2018); Liu L. et al. (2018); Hörner et al. (2019); Hopkins et al. (2021); Duan et al. (2021); Emig et al. (2022)). Reversibility has also been observed in absorbance measurements with spectrophotometry (Figure 3E). Reversibility of material state transitions enables many interesting applications, for example logic gates, further discussed in Section 5.

With tunable and/or reversible stiffness modulation, microscopic material structure is expected to change. Among many possibilities this has been characterized by measuring changes pore size. Optical imaging of EL222-based OptoGel structure revealed little difference in pore size between light and dark states, despite measurable changes in stiffness (Hopkins et al. (2021)) (Figure 3F). These results suggest that imaging the predominant stiffening mechanisms may require higher magnification, potentially using environmental scanning electron microscopy (ESEM) or cryo-electron microscopy (cryo-EM) coupled with focused ion beam (FIB).

Advancing property and structure changes a step further, a material can undergo phase transition and enter states with new useful functionalities. Many opto-enabled materials have demonstrated light-induced gel-sol phase transitions (Xiang et al. (2020); Narayan et al. (2021); Wang et al. (2017); Lyu et al. (2017); Jiang et al. (2020); Zhang et al. (2015)) with rheology (Figure 3G), some even reversible (Zhang et al. (2015); Lyu et al. (2017)). The transition from gel (solid phase) to sol (liquid phase) is defined when the loss modulus *G* ^″^ exceeds the storage modulus *G*′, and occurred after 1.5 h (UV, around 300 nm) for UVR8-based OptoGels (Zhang et al. (2015)), 17 min (violet light, 405 nm, 468.6 mW ⋅ cm^−2^) for PhoCl-based OptoGels (Xiang et al. (2020)), 2 h for Dronpa-based OptoGels (cyan light, 500 nm, nine 3 W LED beads) (Lyu et al. (2017)). Various compositions of OptoGels with cobalamin-binding domains from *myxococcus xanthus* (MxCBD) and *thermus thermophilus* (TtCBD) undergo gel-sol transitions following white light exposures of 5 min (90 klx) (Wang et al. (2017)), 8 min (35 klx) (Jiang et al. (2020)), 20 min (30 klx) (Wang et al. (2017)), and 30 min (30 klx) (Narayan et al. (2021)). Light-induced phase transitions in OptoGels further extends the utility of these materials to applications such as cell elution, further discussed in Section 4.3.

OptoGels have also exhibited non-Newtonian behaviors including shear-thinning, which is a viscosity reduction under increased shear rate (Figure 3H). Shear-thinning was reported for OptoGels composed of Dronpa optoproteins conjugated to PEG networks (Wu et al. (2018)). Optogels consisting of crosslinked optoproteins without additional polymer networks also exhibit shear-thinning, such as Mx/TtCBD-derived optoproteins in SpyTag-SpyCatcher systems (Jiang et al. (2020)). Shear-thinning behavior is particularly valuable for applications involving extrusion-based 3D printing, injectability, and 3D cell culture.

#### 2.2.1 Unexplored Characteristics

The richness and complexity of opto-enabled materials give rise to highly dynamic, non-linear, rate- and time-dependent properties. Exploring material and system properties of soft opto-enabled materials like OptoGels will increase their utility in a variety of engineering and biomedical applications. Many OptoGel material properties remain underexplored or entirely unknown, including: deformation, yielding, plasticity, creep, failure, fracture, and self-healing. Ripe areas of scientific exploration also include system properties of OptoGels, such as: light-programmable friction, lubrication, adhesion, and wear. All of the aforementioned properties would play crucial roles in potential biomedical applications involving OptoGel-cell interfaces (e.g., 3D culture, implants, wound dressings). Knowledge of opto-enabled materials and system properties may inspire new avenues of research and applications.

## 3 Enabling Technologies

There are three main building blocks in what we call an OptoGel: a photoswitchable protein, a conjugation system, and a polymer network, with a few exceptions. Optoproteins have been incorporated into networks with synthetic polymers (Hörner et al. (2019); Wu et al. (2018); Liu L. et al. (2018); Xiang et al. (2020); Hammer et al. (2020); Emig et al. (2022)), natural polymers (Zhang et al. (2015); Hopkins et al. (2021); Narayan et al. (2021)), and optoprotein-based OptoGels without additional polymer networks (Duan et al. (2021); Wang et al. (2017); Lyu et al. (2017); Jiang et al. (2020); Yang et al. (2022)). Conjugation chemistries that do not disrupt optoprotein function make combinatorial assembly efficient. Together, these three building blocks make for a large set of possible materials. In this section, we highlight each of the building blocks for OptoGels. These include optoproteins, conjugation chemistries, and polymer networks.

### 3.1 Optoproteins

OptoGel properties strongly depend on the photoswitch behavior of the incorporated protein. Optoproteins have a myriad of distinct molecular rearrangements in their structure in response to light. Photocaging (Figure 1E) through engineered variants of the j*α* helix of the LOV domain, which locally unfolds in response to light can target a specific protein to be activated (Wu et al. (2009b); Baumschlager and Khammash (2021)). On the other hand, light-induced assembly mechanisms such as dimerization, oligomerization, or dissociation (Figure 1E) (Mansouri et al. (2019); Levskaya et al. (2009); Kennedy et al. (2010); Lu et al. (2020)), as well as photocleavage (Zhang et al. (2017)) can change the cross-linking strength and number when embedded into a polymer matrix. Some optoprotein families (e.g. CBDs and Phytochromes) also need a co-factor, a molecule in addition to the protein itself, present to be able to photoswitch. Overall, the variety of light-response mechanisms in optoproteins widens the spectra of material properties in OptoGels.

Multiple optoproteins’ functions and properties have been studied and are known. The most highly studied, experimentally vetted optoproteins that have been successfully used in non-neuronal optogenetics are phytochromes, cryptochromes, and LOVs (light, oxygen, voltage) (Tischer and Weiner (2014); Lu et al. (2020)). For example, the LOV-domain EL222 optoprotein is sensitive to 450 nm blue light. Illumination generates internal protein-flavin photochemical bonds that in turn induce conformational change of the protein structure, enabling a now free 4*α*-helix to take part in homodimerization with another activated EL222 protein (Nash et al. (2011)). This mechanism was used to reversibly tune the stiffenss of natural collagen hydrogels (Hopkins et al. (2021)). Other properties, like mode of action, photoswitch wavelengths, excitation and reversion times (Figure 2) are well-documented for many optoproteins, and aggregated on the OptoBase platform (Kolar et al. (2018)).

Two especially interesting optoprotein families are CBDs and Phytochromes because they enable multistate control. While most optoproteins have one co-factor, the CBD optoprotein can utilize multiple candidates (e.g. AdoB_12_, MeB_12_, CNB_12_). In a recent paper CBD-based optoproteins were engineered by making a split variant to be tuned to stiffen or soften the OptoGel by illumination depending on the co-factor (Yang et al. (2022)). Phytochromes on the other hand are of particular interest for their multichromatic state control. While many optoproteins revert to their photo-relaxed state in darkness, some phytochroms photoswitch with one wavelength of light and revert with another. Cph1 OptoGels exhibit “triple state” light control to stiffen (660 nm light), soften (740 nm light) (Hörner et al. (2019)), and retain pre-programmed mechanical properties in darkness up to several days (Mailliet et al. (2011)). Furthermore, Cph1 OptoGel stiffness can be finely tuned using the ratio of 660 and 740 nm (Hörner et al. (2019)). This two-wavelength-dependence for photoswitch and reversion is not only a feature of phytochromes but also of cyanobacteriochromes and some fluorescent proteins. Thus, multistate control OptoGels can be achieved by a variety of different ways in their broad design space.

### 3.2 Conjugation Chemistries

Crosslinkers impart form on materials. Conjugation chemistries enable functionalization across a wide range of synthetic and natural polymers, as seen in different OptoGel compositions (Figure 2). Here we review the most commonly used conjugation systems.

#### 3.2.1 SpyTag and SnoopTag

SpyTag-SpyCatcher (2012) (Zakeri et al. (2012)) and the similar SnoopTag-SnoopCatcher (2016) (Veggiani et al. (2016)) systems are designed to link proteins together into multi-protein complexes. The Snoop-system is an orthogonal system to the Spy-system, allowing construction of controlled protein complexes with multiple components (Hatlem et al. (2019)). The SpyCatcher, a modified domain from a surface protein, and the peptide SpyTag recognized by the Catcher, are genetically fused to proteins pre-purification, allowing covalently linked proteins (Hatlem et al. (2019)). Thus, Spy/SnoopTag systems may not be ideal for involving a synthetic polymer but allow optoprotein-based OptoGels without additional polymer networks (Duan et al. (2021); Wang et al. (2017); Lyu et al. (2017); Jiang et al. (2020); Yang et al. (2022)).

#### 3.2.2 Thiol-Maleimide Coupling Reactions

Maleimides react with thiol groups (-SH) and form thioeter-coupled products at pH 6.5–7.5 (Smyth et al. (1964)). Thiols are present on cysteine amino acids when disulfur bonds (S-S) break, making this reaction attractive for protein conjugation. Both Dronpa145-and PhoCl-based OptoGels used solvent-exposed cysteines to bind maleimide groups on functionalized PEG (Wu et al. (2018); Xiang et al. (2020)). PhoCl optoproteins have also been engineered to tune the number of solvent-exposed cysteines to create both softening and stiffening OptoGels from identical illumination conditions (405 nm) (Xiang et al. (2020)).

Thiol-maleimide coupling reactions were also involved in OptoGel conjugation using SNAP-tag. The SNAP-tag (not to be confused with the Snoop- or SpyTags) is a promising general click-chemistry module. It is based on a relatively small 20 kDa protein, and was originally invented to track protein location, timing, and interactions (Keppler et al. (2002)). It binds covalently and irreversibly to benzylguanine (BG) derivatives (Keppler et al. (2002)), and has orthogonal systems available, such as the HALO- (Los et al. (2008)), CLIP- (Gautier et al. (2008)), MCP-, and ACP-tag (Callegari et al. (2012)). Natural polymers that are extracellular matrix (ECM) proteins have large numbers of cysteines (e.g., human collagen Col1A1 has 18 cysteines), which can be conjugated to optoproteins via benzylguanine-maleimide (BG-Mal) and then clicked to SNAP-tags (Hopkins et al. (2021)). The SNAP-tag avoids binding to amides which are necessary for cell-substrate interactions. The SNAP-tag allows essentially any natural or synthetic polymer with functional groups to incorporate optoproteins in OptoGels.

#### 3.2.3 Other Conjugation Chemistries Used in OptoGels

Multiple other conjugation chemistries have been reported in the literature. Cph1-based OptoGels used cysteine thiols to bind vinyl-sulfone (VS.) groups on functionalized PEG (Hörner et al. (2019); Emig et al. (2022)). UVR8-based OptoGels used hexa-peptides to bind tax-interacting proteins on supramolecular nanofibers (Zhang et al. (2015)). LOV2-based OptoGels used strain-promoted azide-alkyne cycloaddition (SPAAC) to bind to tetrabicyclononyne-functionalized PEG (Liu L. et al. (2018)). Many other possible conjugation chemistries for OptoGels are likely to exist and/or are currently being developed, but fall outside the scope of this review.

### 3.3 Polymer Networks

There are two main categories of polymer networks: natural polymers isolated from animal tissues or plants, and synthetic polymers, both which have been incorporated in OptoGels (Figure 2). The main advantages of natural polymer networks over synthetic analogs include their low toxicity and increased biocompatibility (Peppas et al. (2006)). Several biopolymers derived from mammalian ECM have been successfully used to create bio-hydrogels, including collagen, hyaluronan (HA), and fibrin. These biopolymers contain bioactive components that can interact with living cells (Nicolas et al. (2020); Peppas et al. (2006); Stowers (2022)). Agarose and alginate, which are derived from marine algae (Peppas et al. (2006)), are other common bio-hydrogels frequently used in 3D cell culture. While natural polymer networks are more biocompatible than many synthetic polymer systems, they often have higher sample-to-sample variability, less mechanical strength, and can exhibit aging or denaturation over time (Reddy et al. (2021)). To date, three types of natural polymers have been used to create OptoGels: collagen, silk-elastin-like proteins (SELP), and peptide-based nanofibers.

Multiple OptoGels have incorporated synthetic polymer networks (Figure 2). Synthetic polymers have the primary advantage of tunable mechanical properties, also allowing control of transport through the material by regulating mesh size or binding affinity in the polymer network. Unlike natural polymers, synthetic analogs cannot provide biochemical cues to facilitate cell signaling; however, signaling molecules such as peptides, growth factors, and glycans can be incorporated within OptoGels to recapitulate natural extracellular environments (Nicolas et al. (2020)). Synthetic polymer networks that could be good candidates for future opto-enabled materials include several that are currently used in hydrogels for biomedical applications: polyethylene glycol (PEG), polyvinyl alcohol (PVA), polyhydroxyethylmethacrylate (pHEMA), polyacrylamide (PAAm), and poly (N′-isopropylacrylamide) (pNIPAm) (Ahmad et al. (2022)). However, among these polymer networks only variants of PEG have been used in OptoGels.

Polyethylene glycol (PEG) is one of the most widely used synthetic polymers in biomedical applications, and is known for its non-toxicity and ease of functionalization (Peppas et al. (2006)). OptoGels have used multiple variants of PEG networks (Figure 2). Outside of OptoGel systems, PEG networks have been tuned to achieve large ranges in stiffness and permeability, depending on crosslink density and mesh size, which is defined as the average spacing between all neighboring polymer chains in a 3D network (De Gennes and Gennes (1979); Kyburz and Anseth (2015)). In good agreement with scaling concepts from polymer physics (De Gennes and Gennes (1979)), increasing PEG concentration has been shown to decrease hydrogel mesh size, swelling, and permeability (Cruise et al. (1998); Bal et al. (2014); Zustiak and Leach (2011); Sokic and Papavasiliou (2012); Phelps et al. (2012)), while increasing stiffness (Richbourg and Peppas (2020); Weber et al. (2009); Bal et al. (2014); Zustiak and Leach (2011); Sokic and Papavasiliou (2012)). Bulk PEG hydrogels with increased molecular weights have been correlated with increased swelling (Keys et al. (1998); Metters and Hubbell (2004)), as well as increased strain to failure and decreased stiffness (Yang et al. (2011)). Crosslink functionality is tuned by the number of arms on star-PEG polymers. Keeping arm molecular weight constant, increased arm number has been correlated with decreased swelling (Kim et al. (2016)) and permeability (Lee et al. (2016)). Each arm of a star-PEG network may have functional end-groups; the reaction kinetics during PEG hydrogel formation may be affected by end-group chemistry (Kim et al. (2016)). Thus, multiple parameters to control properties of a PEG network are known, implicitly facilitating OptoGel design towards desired material properties.

## 4 Emerging Applications

### 4.1 OptoGels in Mechanobiology

The extracellular matrix (ECM) surrounding all of the cells in our body is composed of >300 distinct proteins, and forms an organized 3D structure (Nicolas et al. (2020)). Each unique tissue in the body is supported by a specific set of organized ECM components, endowing every organ with unique mechanical characteristics (Yeung et al. (2005)). In recent years it has become clear that the mechanincal and chemical cues transmitted through the ECM can regulate a wide range of cell behaviors ranging from proliferation, to differentiation, to adhesion and spreading (Hynes (2009)), giving rise to the booming field of Mechanobiology.

Despite the success of Mechanobiology studies, many open questions regarding the complex communication mechanisms between cells and their local mechanical environment remain unanswered (Nicolas et al. (2020)). ECM structure and composition are not static, highlighting the need for dynamic ECM mimicking materials (Nicolas et al. (2020); Hynes (2009)). Thus, a class of questions revolving around the dynamics of the mechanobiological response can be addressed through engineered ECM materials with light programmable mechanochemical properties. For example, how do mechanosignaling systems quantitatively sense fluctuating environmental mechanics? How are cellular mechanical memories established, and how long do they last? What are the specific features of a dynamic mechanical stimulus that are sensed by cells (e.g. tension, compression, shear, stress relaxation)?

Existing OptoGel platforms benefit from exquisitely precise and reversible tunability of stiffness allowing researchers to quantitatively probe mechanosignaling mechanisms. Indeed, other non-optoprotein-based tunable hydrogels platforms have been used to probe these questions. For example, the control parameters of pH- and temperature-responsive gels (Klouda and Mikos (2008); Ahiabu and Serpe (2017); Bratek-Skicki (2021)) are not orthogonal to the cellular sensory network. In contrast, ambient light levels required to tune OptoGel mechanics do not alter cellular gene expression (Hörner et al. (2019)). In addition, pH and temperature have limited spatial control while light patterning has the advantage of having sub-micrometer spatial resolution (Johnson et al. (2017)).

In a landmark study, Hörner *et al.* demonstrated the utility of OptoGels in probing cellular mechanobiology with their 8-armed PEG-based, red/infrared light responsive OptoGel. The dual wavelength-responsive Cph1 optoprotein can be activated with red light (660 nm) and deactivated in IR (740 nm). These Cph1-based OptoGels can be tuned by varying the ratio of red:IR light. Due to very low levels of phototoxicity of such low-energy wavelengths and the relatively long half-life of Cph1 photo-activated states, Hörner *et al.* were able to probe the gene regulatory response to ECM stiffening using bulk RNA sequencing (Hörner et al. (2019)).

Other studies have begun to apply OptoGels to understand the role of dynamical mechanical cues on cell migration and differentiation, taking advantage of the OptoGels’ spatiotemporal control. Nanoindentations of cells grown on Cph1-based OptoGels under different illumination conditions showed that both storage and loss moduli of the underlaying gel influence cell stiffening (Emig et al. (2022)). Dronpa-based OptoGels have been used to dynamically control cell migration rates by tuning substrate stiffness; increasing ECM crosslink density may speed up wound healing (Wu et al. (2018)). Others have found that cyclic light-induced stiffening and softening increase rates of fibroblast-to-myofibroblast transformation than for constantly stiffening OptoGels (Liu L. et al. (2018)). Together, these studies highlight the promise of dynamically tunable OptoGels for dissecting mechanosignaling mechanisms and applying them as non-invasive methods for reprogramming cell states *in vitro*.

### 4.2 OptoGels in 3D Cell and Organoid Culture

OptoGels are promising tools for scaling clinically viable cell cultures and organoids, which are miniature *in vitro* tissue approximations for *in vivo* organs (Kim et al. (2020); Heo et al. (2022)). While nearly all cells of our bodies develop in a 3D environment, multiple cell culture techniques are still 2D, likely due to the ease of passaging and imaging monolayers. However, cells expanded in 2D (notably stem cells (Tian et al. (2009); Baharvand et al. (2006); Zhou et al. (2017); Liu C. et al. (2018))) exhibit significant differences in gene expression and even long-term functional capabilities compared to cells expanded in 3D (Pampaloni et al. (2007); Kyburz and Anseth (2015)). A major challenge associated with growing cells in 3D is harvesting at scale. Thus, optogels that can reversibly cross the gel-sol transition to enable non-destructive harvesting of therapeutic cells poses an exciting and unique application for light-based dynamical control of the ECM.

The gold standard scaffold for generating organoids is Matrigel, a passive gel whose properties can be neither spatially nor dynamically controlled (Heo et al. (2022)). Thus, organoid model systems can suffer from a lack of reproducibility (Gjorevski et al. (2022)), which is especially problematic in clinical adaptions where reproducible organoid patterning will be essential. Excitingly, even the most basic manipulation of Matrigel (e.g., adding it on top of patterned 2D stem cell monolayers) has profoundly advanced our ability to engineer organoid structure and function (Karzbrun et al. (2021)). In addition, recent studies showed that spatiotemporal control through a light-degradable hydrogel that contained a combination of mechanically static PEG and hydrolytically light-degradable PEG could be used to determine organoid structure (Gjorevski et al. (2022)). Thus OptoGels, with their exquisite spatiotemporal control, have enormous potential for both increasing the reproducibility of organoids and also engineering structures with complex geometries.

### 4.3 Programmable Cell and Protein Eluting Materials

Engineered polymers for controlled release of small molecule drugs revolutionized medicine in the late 1970s (Langer and Folkman (1976)). OptoGels could provide a programmable elution platform for future cell therapies and biologics. Light-induced gel-sol transition has the potential for controlled release of cells in the body (Xiang et al. (2020); Narayan et al. (2021); Wang et al. (2017); Lyu et al. (2017); Jiang et al. (2020); Zhang et al. (2015)). In these platforms the cell-eluting matrix releases cells by melting away (Zhang et al. (2015); Wang et al. (2017); Jiang et al. (2020); Narayan et al. (2021)). However, for such applications light toxicity should be considered in terms of wavelength penetrance, energy, intensity, and exposure time, previously discussed in Section 2.2. Light delivery could happen through the skin using red/IR wavelengths, upconversion particles embedded in the tissue, or implanted fiberoptics as is routinely done in neuronal optogenetic systems. These systems could be endowed with feedback control to ensure optimal release times and avoid unwanted side effects, such as the toxic shock seen in immunotherapy trials (Schirrmacher (2019); Arroyo-Currás et al. (2018)). Thus, OptoGels with their light-programmable control of, even reversible, gel-sol transitions, hold promise for the programmable release of cells.

OptoGels also have potential in providing controlled release of biologics. Potential applications are manyfold ranging from spatiotemporal release of ligands in a wound to directing the differentiation of stem cells into designer tissues. Protein release mechanisms, when paired with holography or 2-photon stimulation could even be used to direct biochemical stimuli in a 3D cell culture (Nicolas et al. (2020)). Indeed, OptoGels have been reported to spatiotemporally deliver proteins, such as Zdark (Hammer et al. (2020)) or GFP (Zhang et al. (2015); Jiang et al. (2020)). Thus, protein-eluting OptoGels have potential applications in directing cells either in or on the human body or in an *in vitro* tissue engineering context.

## 5 Discussion and Future Prospects

We foresee a range of potential applications that have yet to be explored. In particular, wound healing topicals and wearable prosthetics that leverage OptoGels’ dynamic control could be programmed, with light, to be more responsive to the needs of the human body. OptoGels designed with cell-signaling control to guide migrating cells and mitigate negative inflammatory cues could one day be incorporated into advanced bandages. Sensing/responding-functionalities could help usher in disposable, soft robotics platforms into wearable sensors, such as contact lenses or prosthetics. Surface-conjugation of optoproteins for reversible, biodegradable, and photoswitchable adhesives could have applications for delicate or vulnerable skin, especially important in neonatal and geriatric care. Indeed, their potential may only grow as we exploit the vast design space, as well as reveal yet unexplored physical properties and characteristics.

A large unexplored design space does not only exist for opto-enabled materials, but also for optoproteins themselves, and their respective responses to light stimuli. Some optoproteins convert light energy to mechanical energy, as in the case of photocaging, dimerization, oligomerizaton, and dissociation mechanisms. In contrast, opsin-based proteins, briefly discussed in Section 2, instead have an ion-pump function, converting light energy to chemical energy. What other protein-encoded, photon energy conversion mechanisms has nature evolved? Only a handful of optoproteins have been purified and examined *in vitro*, while thousands of optoproteins have been detected through genome sequencing (Glantz et al. (2016)). Do proteins that directly convert light into thermal or electrical energy exist? If so, these may be leveraged to power next-generation biosensors. In addition, we observe that multiple photo-responsive domains are clustered together on the same polypeptide suggesting that these proteins require the absorption of multiple photons of the same wavelength or are detecting the coincidence of multiple wavelengths to actuate their signals (Burgie and Vierstra (2015)). Such natural architectures are reminiscent of logic gates like AND, OR, NOT. In a recent work, a computational system decoding the number of input light pulses applied was designed from a polymer network with incorporated optoproteins (Beyer et al. (2018)). Future applications may take nature’s lead and engineer logic directly into material matrices.

Nascent interdisciplinary fields can be accelerated by having a common place to gather. Bell Laboratories was remarkable for gathering the materials scientists, atomic physicists, and theorists in the same building to jumpstart the semiconductor industry. In contrast, the present OptoGel field is scattered. Thus far, 13 OptoGels have been reported in 12 different journals (Table 1). We hope this review provides a new space for this budding field by illuminating the connections and synergies between extracellular optogenetics at the interface of synthetic biology and materials science.

**TABLE 1:** List of manuscripts for OptoGels presented in Figure 2.

Author	Year	Title	Journal
Zhang et al	2015	Rational design of a photo-responsive UVR8-derived protein and a self-assembling peptide–protein conjugate for responsive hydrogel formation	Nanoscale
Wang et al	2017	B12-dependent photoresponsive protein hydrogels for controlled stem cell/protein release	Proceedings of the National Academy of Sciences
Lyu et al	2017	Optically controlled reversible protein hydrogels based on photoswitchable fluorescent protein Dronpa	Chemical Communications
Liu et al	2018	Cyclic Stiffness Modulation of Cell-Laden Protein-Polymer Hydrogels in Response to User-Specified Stimuli including Light	Advanced Biosystems
Wu et al	2018	Reversible hydrogels with tunable mechanical properties for optically controlling cell migration	Nano Research
Hörner et al	2019	Phytochrome-Based Extracellular Matrix with Reversibly Tunable Mechanical Properties	Advanced Materials
Xiang et al	2020	Hydrogels With Tunable Mechanical Properties Based on Photocleavable Proteins	Frontiers in Chemistry
Jiang et al	2020	Injectable, photoresponsive hydrogels for delivering neuroprotective proteins enabled by metal-directed protein assembly	Science Advances
Duan et al	2021	Light-Responsive Dynamic Protein Hydrogels Based on LOVTRAP	Langmuir
Hopkins et al	2021	An Optogenetic Platform to Dynamically Control the Stiffness of Collagen Hydrogels	ACS Biomaterials Science and Engineering
Narayan et al	2021	Dynamically tunable light responsive silk-elastin-like proteins	Acta Biomaterialia
Yang et al	2022	B12-induced reassembly of split photoreceptor protein enables photoresponsive hydrogels with tunable mechanics	Science Advances
Emig et al	2022	Benchmarking of Cph1 Mutants and DrBphP for Light-Responsive Phytochrome-Based Hydrogels with Reversibly Adjustable Mechanical Properties	Advanced Biology
